# Nitrogen-Rich Porous Organic Polymers from an Irreversible Amine–Epoxy Reaction for Pd Nanocatalyst Carrier

**DOI:** 10.3390/molecules28124731

**Published:** 2023-06-13

**Authors:** Ailing Li, Fuping Dong, Yuzhu Xiong

**Affiliations:** Department of Polymer Materials and Engineering, College of Materials and Metallurgy, Guizhou University, Guiyang 550025, China; ailing_li2023@126.com

**Keywords:** porous organic polymer, amine–epoxy, ring opening, catalyst carrier

## Abstract

Nitrogen-rich porous organic polymers were fabricated through a nonreversible ring-opening reaction from polyamines and polyepoxides (PAEs). The epoxide groups reacted with both primary and secondary amines provided by the polyamines at different epoxide/amine ratios with polyethylene glycol as the solvent to form the porous materials. Fourier-transform infrared spectroscopy confirmed the occurrence of ring opening between the polyamines and polyepoxides. The porous structure of the materials was confirmed through N_2_ adsorption–desorption data and scanning electron microscopy images. The polymers were found to possess both crystalline and noncrystalline structures, as evidenced by X-ray diffraction and high-resolution transmission electron microscopy (HR-TEM) results. The HR-TEM images revealed a thin, sheet-like layered structure with ordered orientations, and the lattice fringe spacing measured from these images was consistent with the interlayer of the PAEs. Additionally, the selected area electron diffraction pattern indicated that the PAEs contained a hexagonal crystal structure. The Pd catalyst was fabricated in situ onto the PAEs support by the NaBH₄ reduction of the Au precursor, and the size of the nano-Pd was about 6.9 nm. The high nitrogen content of the polymer backbone combined with Pd noble nanometals resulted in excellent catalytic performance in the reduction of 4-nitrophenol to 4-aminophenol.

## 1. Introduction

Porous materials, with their remarkable physical and chemical properties and intricate pore structures, have been extensively employed in fields such as catalysis, sensing, adsorption, energy storage, and biomedicine [1,2]. The development of porous materials has evolved from natural resources to artificial synthesis, from inorganic to organic–inorganic hybrid, and finally to organic porous materials [3]. Porous organic polymers consist of a network structure of organic building blocks connected by covalent bonds. Organic porous polymers offer several advantages over traditional inorganic porous materials, such as a lower density due to the lighter elements they are composed of (C, H, O, N, B, etc.), adjustable sizes of their building blocks, and multifunctionality [4]. Furthermore, they are highly stable in different solvents, such as acidic, alkaline, water, and regular organic solvents, and can remain stable at both room and higher temperatures due to their covalently bonded high-polymer network structure [5,6]. This makes them particularly useful as their chemical composition and porous structure can be tailored to meet specific requirements.

The incorporation of heteroatoms or groups containing heteroatoms, such as nitrogen, boron, sulfur, and amino, nitro, and sulfonic acid, into porous organic polymers has been demonstrated to enhance their performance in various aspects [7]. By constructing porous organic materials with high nitrogen content or nitrogen-containing functional groups, a variety of nitrogen-rich porous organic polymers can be fabricated [8,9]. These polymers exhibit strong adsorption for Lewis acid molecules and increased catalytic activity due to electron transfer in the π condensed system. Additionally, they demonstrate improved hydrophilicity and high affinity for metal ions, making them suitable materials for catalyst carriers. Compared to other materials, such as MOFs [10], nitrogen-containing porous organic polymers are more resistant to water and oxygen, and are not prone to collapse in certain solvents, making them more economical and environmentally friendly [11].

Nitrogen-rich porous organic polymers have emerged as a promising carriers for noble metal nanoparticles as catalysts [12,13] due to their excellent pore structure, including adjustable pore size and high surface area [14], as well as their high nitrogen content, such as –NH_2_ and –NH– groups, which have an affinity for noble metals [15]. Common nitrogen-rich porous organic polymers are based on triazines, heptazines, porphyrins, and carbazoles [16,17]; however, some of these polymers are covalent organic frameworks with crystalline structure, which are unstable under certain conditions [18,19]. Initially, reversible reactions were used to construct polymers with crystalline structures, as the reversible process facilitates the self-repair of the materials to form a more complete crystal [20,21,22,23]. However, there is an inverse relationship between crystallinity and stability, where increasing the crystallinity leads to a decrease in stability and vice versa [24]. Therefore, several successful strategies have been developed to increase both crystallinity and stability of the materials simultaneously. 

At present, there are two potential strategies for addressing problems: one still involves reversible reactions, whereby the bonding is modified, such as the conversion of keto-enol tautomers and imine bonds, introducing hydrogen bonds or other interaction forces to enhance the stability of materials, and constructing interlayer chemical links [25]. The second approach involves the utilization of irreversible bonding techniques to fabricate materials. This can be achieved through the synthesis of ether linkages using nucleophilic aromatic substitution reactions [26], as well as the preparation of C–C linkages via Suzuki reactions [27]. Additionally, the emergence of nonreversible reactions offers possibilities for the construction of porous polymers that demonstrate remarkable stability and crystalline structure. The production of novel nitrogen-rich porous organic polymers with high stability through nonreversible polymerization is an urgent requirement.

This paper presents a novel class of nitrogen-rich materials, referred to as PAEs, which possess polyhydroxyl and polyimino functional groups. These materials were synthesized through the ring-opening reaction of polyamines and polyepoxides, without the use of any catalyst. The fabrication process involved the polymerization of tris(4-hydroxyphenyl)methane triglycidyl ether (TGTPM) and N′,N′-bis(2-aminoethyl)ethane-1,2-diamine (TREN), with polyethylene glycol (PEG) serving as a solvent and porogen. The resulting polymers contain numerous N-rich functional groups, such as amino/imino groups, and exhibit a high affinity for metals. The stability of the PAEs in water and organic solutions was also investigated. Furthermore, the PAEs were utilized as carriers to immobilize Pd nanoparticles, which demonstrated exceptional catalytic performance in the reduction of 4-nitrophenol (4-NP) to 4-aminophenol (4-AP). These nitrogen-rich porous organic polymers synthesized in a straightforward process exhibit excellent chemical/thermal stability, strong affinity for metals, and high potential for application in a variety of fields, particularly in catalysis.

## 2. Results and Discussion

**Figure 1:**
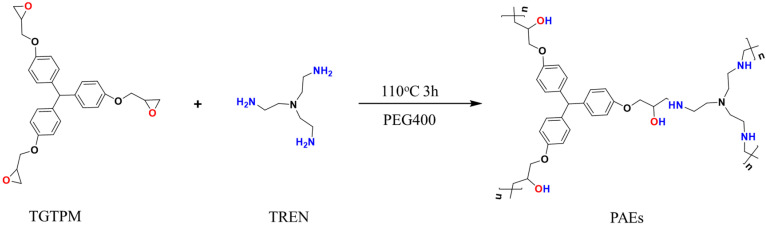
Synthetic scheme of porous amine–epoxy (PAE) polymers.

**Figure 2:**
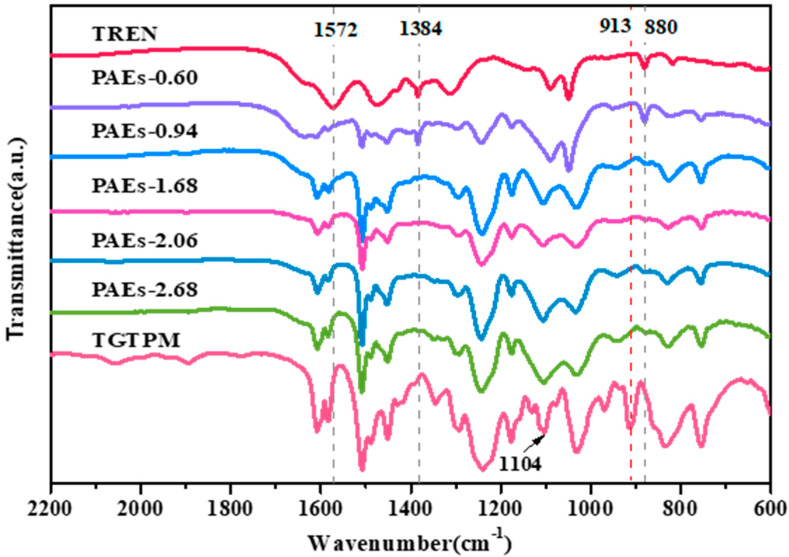
FT-IR spectra of TREN, TGTPM, and PAEs (PAEs-X, X = 0.60, 0.94, 1.68, 2.06, 2.68).

The porous polymers with a high nitrogen content were fabricated through the ring-opening reaction of polyepoxides and polyamines, utilizing PEG as a solvent and operating at a temperature of 110 °C. The scheme for the synthesis of the porous materials and the hypothetical general structure is presented in Figure 1. The ring opening of epoxy groups with primary amine groups was confirmed by Fourier-transform infrared spectroscopy (FT-IR). The FT-IR spectra of the monomers and the PAE polymers synthesized with varying polyepoxide-to-polyamine mole ratios were analyzed, as shown in Figure 2. The disappearance of the N–H bending at 1572 cm^−1^ for the primary amine and the C–O–C band at 913 cm^−1^ for epoxide in all polymer spectra indicated the formation of the polymers and the depletion of the monomers [28]. At a triepoxide-to-triamine mole ratio of 0.60, which indicates an abundance of amine, the final reaction product still contained the primary amine group, as evidenced by the vibration and deformation of C–N groups at 1384 and 880 cm^−1^ [29]. This observation suggested that the reaction was incomplete, which was further supported by the oil-like state of the final sample. For other polymers with epoxide/amine ratios ranging from 0.94 to 2.68, the ring opening between polyamine and polyepoxide was confirmed by the absence of peaks at 1384 and 880 cm^−1^ for primary amine and at 913 cm^−1^ for epoxide [30].

**Figure 3:**
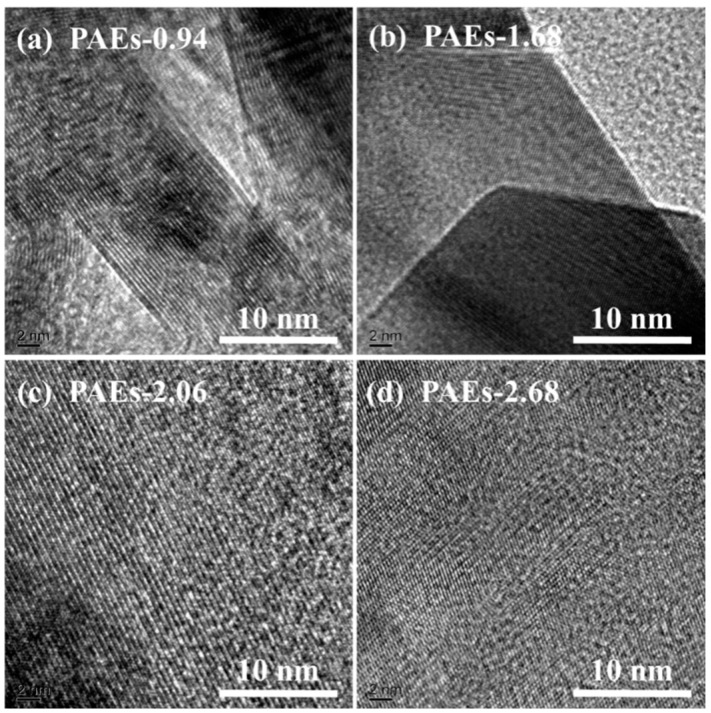
HR-TEM images of PAEs (PAEs-X, X = (**a**) 0.94, (**b**) 1.68, (**c**) 2.06, (**d**) 2.68).

**Figure 4:**
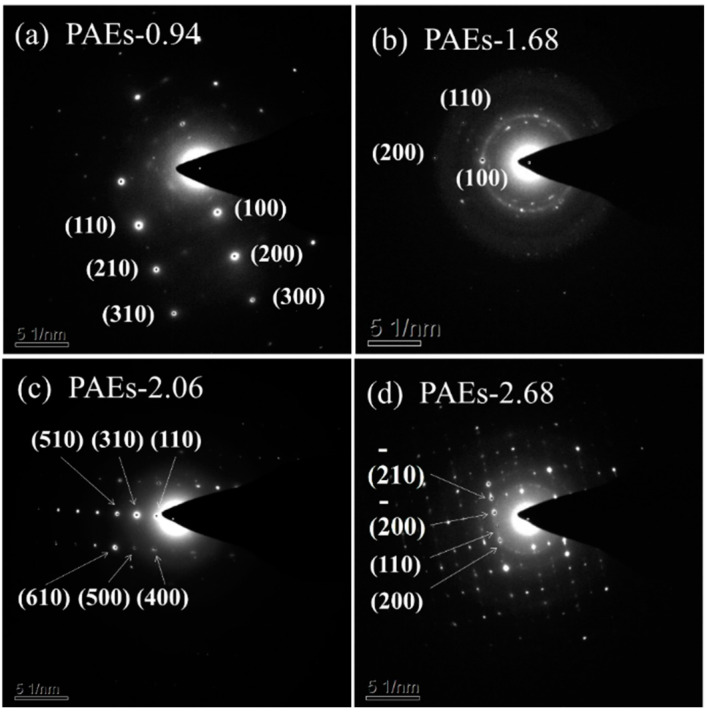
Electron diffraction patterns of PAEs (PAEs-X, X = (**a**) 0.94, (**b**) 1.68, (**c**) 2.06, (**d**) 2.68).

The material’s crystal structure was examined through the utilization of high-resolution transmission electron microscopy (HR-TEM) and powder X-ray diffraction (PXRD) techniques. The HR-TEM image (Figure 3) reveals that the crystal structure morphology of all PAE polymers is noticeably regular and clear, exhibiting an ordered layered structure [31,32]. The HR-TEM images reveal that the ordered layered structures in PAE-0.94, PAE-1.68, PAE-2.06, and PAE-2.68 exhibit a spacing of approximately 0.22, 0.23, 0.28, and 0.31 nm, respectively. These structures are located within the distance of aromatic stacking (<0.6 nm) and the range of hydrogen bonding interaction (0.24–0.32). This suggests that the crystallization driving force of PAEs is attributed to the intramolecular and intermolecular hydrogen bonding between hydroxyl and amine functional groups, as well as the π–π stacking effect between benzene rings. The selected area electron diffraction patterns (as shown in Figure 4 and Table 1) of each sample are consistent with the hexagonal crystal system.

**Figure 5:**
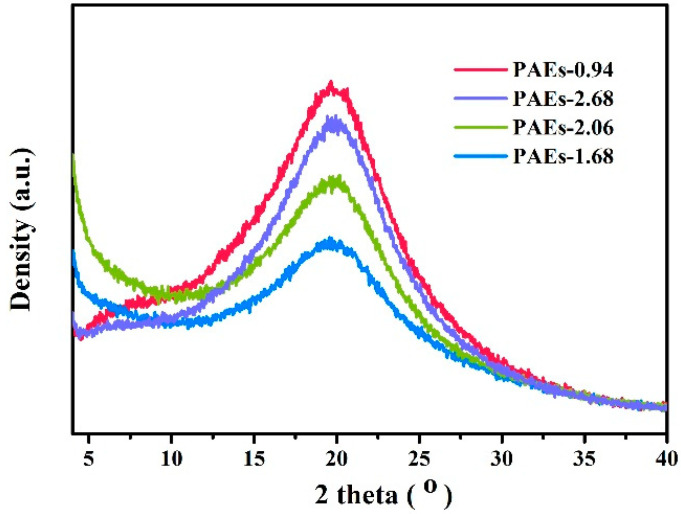
PXRD pattern of PAEs (PAEs-X, X = 0.94, 1.68, 2.06, 2.68).

**Figure 6:**
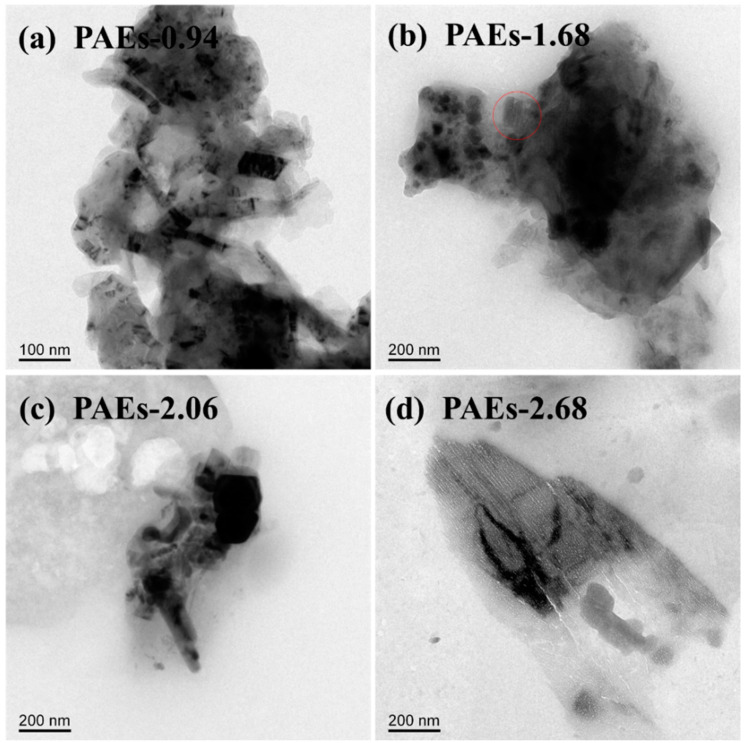
TEM images of PAEs (PAEs-X, X = (**a**) 0.94, (**b**) 1.68, (**c**) 2.06, (**d**) 2.68).

Analysis of the polymer crystal structure using powder X-ray diffraction technology is illustrated in Figure 5. The PXRD spectrum indicates that the material has a certain degree of crystallinity; however, it is not high. This could be due to the grain size being less than 300 nm, which is beyond the testing range of PXRD. Additionally, the PAE-0.94 sample has the narrowest half peak width, indicating that its crystallinity is superior to the other samples. Figure 6 displays the transmission electron microscopy (TEM) diagram of the PAE-X sample. The selected area electron diffraction test discloses that the dark region in the diagram denotes the crystalline area, while the light transparent area is predominantly amorphous in structure. The size of the crystalline part is primarily less than 200 nm, which further substantiates the hypothesis that the grain size exceeds the testing range of PXRD.

**Figure 7:**
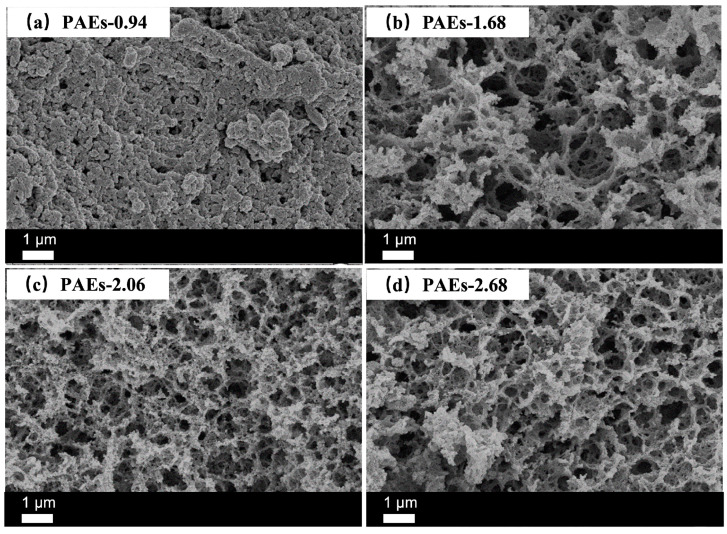
SEM images of PAEs (PAEs-X, X = 0.94 (**a**), 1.68 (**b**), 2.06 (**c**), 2.68 (**d**)).

The cross-sectional samples were analyzed using scanning electron microscopy (SEM, Zeiss, Jena, Germany) to characterize their morphology and microstructure. The ratios of polyepoxide to polyamine were adjusted to 0.94 (excess triamine) and 1.68, 2.06, and 2.68 (excess triepoxide), while the total weight fraction of monomers to PEG was 0.10. The results indicated that the sample exhibited a stacked pore morphology (Figure 7a) at a molar ratio of polyepoxide to polyamine of 0.94, which was significantly different from the other three ratios. However, when the mole ratio increased to 1.68, 2.06, and 2.68, the porous structure was clearly visible (Figure 7b–d). The analysis suggests that the difference in morphology is due to the reaction between the epoxy and primary amino groups. When the epoxy and primary amino groups are equivalent, the reaction tends to open the ring between them, resulting in an ideal honeycomb-like structure with a low degree of intermolecular crosslinking. However, when there is an excess of epoxy, the reaction between the secondary amino group and epoxy, as well as the polymerization of epoxy under alkaline conditions, leads to a higher degree of crosslinking in the material. When the crosslinking degree is low, the pores caused by polymerization-induced phase separation induced by polyethylene glycol are prone to collapse, while at high crosslinking degrees, they can be well maintained, resulting in a loose and porous network structure [33]. The reaction between polyepoxide and polyamine formed gels, while PEG acted as a reaction medium and porogen to form pores after removal.

**Figure 8:**
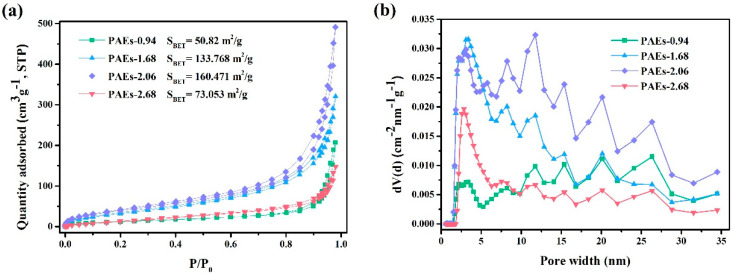
(**a**) N_2_ adsorption isotherms of PAEs (PAEs-X, X = 0.94, 1.68, 2.06, 2.68) at 77 K; (**b**) pore size distribution histogram of PAEs (PAEs-X, X = 0.94, 1.68, 2.06, 2.68) calculated from DFT fitting of the adsorption branch of the N_2_ adsorption isotherm at 77 K.

N_2_ adsorption was utilized to assess the permanent porosity of the polymers (PAE-0.94, 1.68, 2.06, and 2.68). The results shown in Figure 8 demonstrated that all samples exhibited a type II isotherm, which is a key characteristic of porous materials [34]. This isotherm is characterized by the coincidence of the adsorption and desorption curves, forming an S-shape. An inflection point is observed at a low P/P_0_, indicating the saturated adsorption capacity of the monolayer. As the relative pressure increases, the second layer begins to be formed and the number of adsorption layers become infinite at the saturated vapor pressure. The surface areas of the samples were measured as 50.9, 133.8, 160.5, and 73.1 m^2^/g for PAEs-0.94, 1.68, 2.06, and 2.68, respectively. Using the density functional theory (DFT) method, the average pore diameters were calculated as 25.2, 14.8, 18.9, and 12.5 nm for PAEs-0.94, 1.68, 2.06, and 2.68, respectively. Analysis of the pore size distribution reveals a range of random peaks, which are partially attributed to the micropores from the molecular structure and the disordered mesopores formed by PEG.

**Figure 9:**
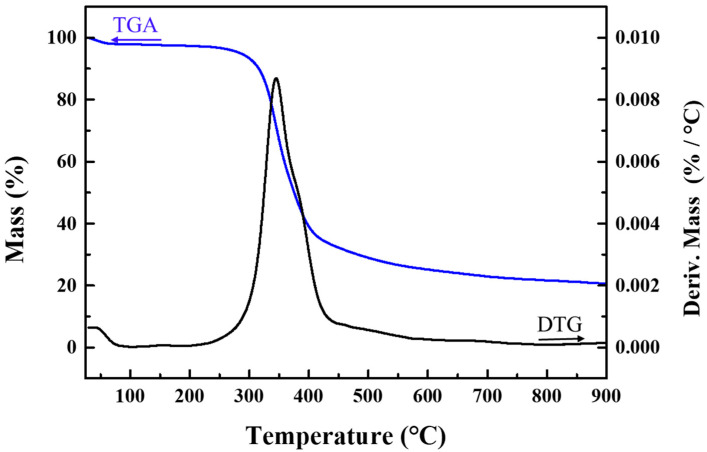
Thermal gravimetric analysis/derivative thermogravimetry curve of PAEs-2.06 under N_2_ atmosphere.

The thermal stability of the polymer was examined through thermal gravimetric analysis/derivative thermogravimetry (TGA/DTG), wherein its weight loss was monitored from room temperature to 900 °C under N_2_ atmosphere. The results, as depicted in Figure 9, indicate that the polymer can withstand temperatures up to 220 °C. The total weight loss rate of the sample is 91.9% from room temperature to 620 °C, during which the fastest weight loss occurs between 300 °C and 620 °C and the weight loss remains unchanged after 620 °C. Apart from its thermal stability, the aromatic polymer also exhibits commendable chemical stability and solvent resistance, as reported in previous studies [35]. The solubility of the PAE polymer was evaluated in various organic solvents, and it was found to be insoluble in methanol, DMSO, THF, and DMF. The excellent chemical and thermal stability of the polymer make it a good choice as a catalyst support for noble nanometals in heterogeneous catalysis.

**Figure 10:**
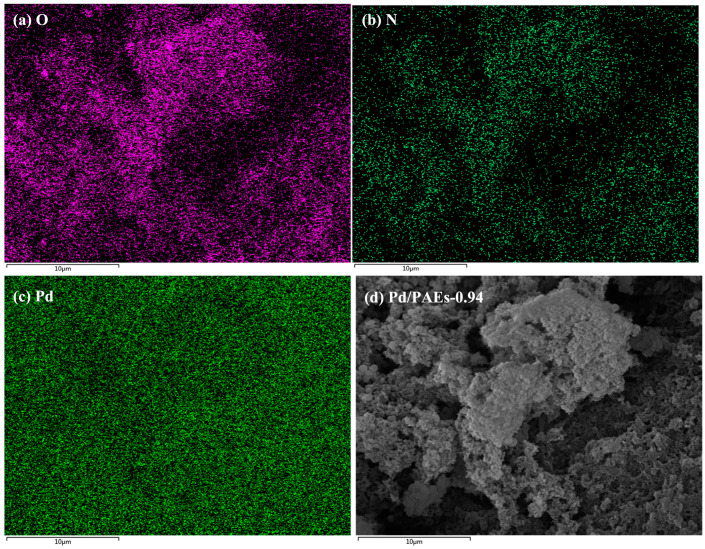
EDX mapping of (**a**) O, (**b**) N, (**c**) Pd element for Pd/PAEs-0.94 and (**d**) the corresponding SEM image for the same area.

**Figure 11:**
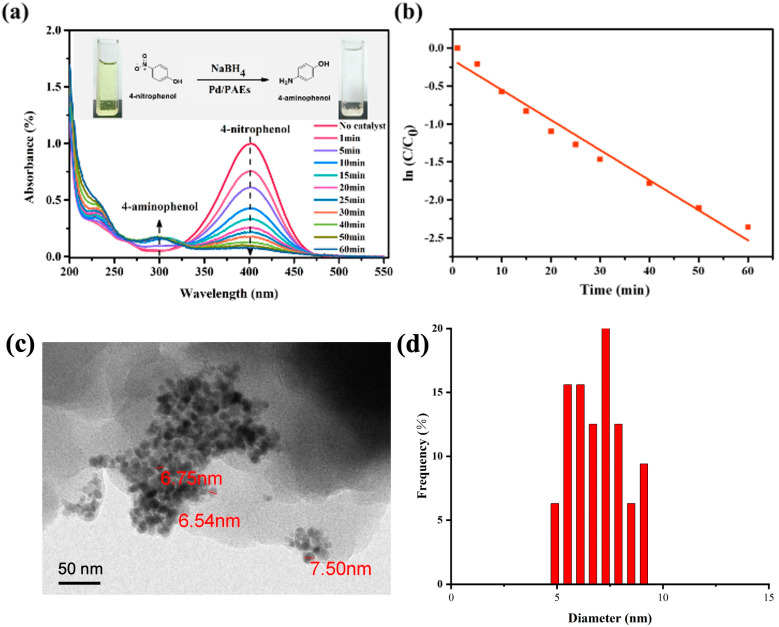
(**a**) UV–Vis spectra of 4-nitrophenol reduction with Pd/PAEs-0.94 hybrid nanocomposites as catalyst; (**b**) quasi-first-order reaction kinetic equation for the degradation of 4-nitrophenol by sample Pd/PAEs-0.94; (**c**) TEM image and (**d**) the corresponding size distribution of the Pd nanoparticles on PAEs.

The porous structure and imino groups present on the surface of the material make it suitable for use as a catalyst carrier, particularly when immobilized with noble metal nanoparticles [36]. The PAEs developed in this study contain numerous electron-rich groups, including imino and hydroxyl groups, which have an affinity for metal ions [37]. The elemental analysis (EA) of sample PAEs-0.94 revealed an actual nitrogen content of approximately 4.3%, which is slightly lower than the theoretical content of 5.1%. Energy-dispersive X-ray spectroscopy (EDX, Zeiss, Jena, Germany) was utilized to confirm the element distribution on the porous polymer material after the immobilization of Pd. EDX mapping revealed an even distribution of oxygen and nitrogen elements in the polymer, as seen in Figure 10a,b. Furthermore, Pd nanoparticles were loaded onto the porous polymer via impregnation–reduction, and EDX mapping confirmed the presence of Pd element on the polymer (Figure 10c). The impregnation–reduction method enables in situ reduction of metal ions, which are then converted into nanoparticles that are affixed to the material surface. This prevents aggregation of metal catalysts and improves their catalytic capability. Transmission electron microscopy (TEM) was employed to verify the presence of Pd nanoparticles on the porous material. As depicted in Figure 11c, the Pd nanoparticles were observed to be loaded onto the polymer surface in a monodisperse manner, with an average particle size of 6.9 nm. According to inductively coupled plasma (ICP) data, the Pd content in the composite was 5.9 wt%.

The immobilization of noble metals on porous organic polymer support is a good approach to improve the stability of nanocatalysts in chemical reactions and reduce the cost of noble metal catalysts. The catalytic performance of the Pd/PAES-0.94 catalyst was evaluated through a model reaction of 4-nitrophenol (4-NP) reduction to 4-aminophenol (4-AP) in water by NaBH_4_, and the reaction process was monitored through ultraviolet–visible spectroscopy. In the absence of a catalyst, no discernible color change was observed, indicating that the reduction of 4-NP did not occur. However, when Pd/PAEs were used as catalysts, the absorption intensity at 400 nm, which is the characteristic peak for 4-NP, decreased progressively over time, and a corresponding new band appeared at 300 nm, indicating the formation of 4-AP (Figure 11a). The presence of an isosbestic point at 325 nm in the ultraviolet–visible spectroscopy (UV–Vis) spectra indicated that the catalytic reduction of 4-NP produced 4-AP without any byproduct formation [38]. The complete conversion of 4-NP was confirmed by the color change of the system from bright yellow to colorless (as shown in the inset of Figure 11a). In the reaction, an excessive amount of sodium borohydride was used, allowing the reaction rate to be approximated as only dependent on the concentration of 4-NP; thus, a quasi-first-order reaction kinetics equation was established. The apparent rate constant (kapp) of the reduction of p-nitrophenol to p-aminophenol at room temperature was found to be 0.07869 min^−1^. The Pd nanoparticles were fixed onto the surface of the PAEs, which prevented the aggregation of the metal catalysts and greatly improved their catalytic ability. 

## 3. Materials and Methods

### 3.1. Reagents

Tris(4-hydroxyphenyl)methane triglycidyl ether (TGTPM) was purchased from Sigma Aldrich Chemicals. *N*,*N*-bis(2-aminoethyl)ethane-1,2-diamine (TREN, 96%), polyethylene glycol (PEG, Mn avg = 400), and palladium chloride (Pd approximately 59–60%) were supplied by Aladdin Reagents Co., Ltd. (Shanghai, China). Sodium borohydride (NaBH_4_, 98%) was purchased from Kermel Reagents Co., Ltd. (Tianjin, China). The 4-nitrophenol (4-NP, 99%) was purchased from Energy Chemical Co., Ltd. (Shanghai, China). Sodium hydroxide (NaOH, AR) was purchased from Sinopharm Chemical Reagents Co., Ltd. (Shanghai, China). Hydrochloric acid (HCl, AR, approximately 36–38%) was purchased from Chuandong Chemical Co., Ltd. (Chongqing, China).

### 3.2. Preparation of PAEs

A range of polyamide–epoxides (PAEs) were synthesized by utilizing varying polyepoxide-to-polyamine mole ratios, namely, 0.60, 0.94, 1.68, 2.06, and 2.68. PEG was employed as the solvent, and the total weight proportion of monomers in PEG was 0.10. Around 1.0 g of TGTPM (triepoxide, E3, 2.17 mmol; 6.51 mmol epoxy groups/g) monomer was dissolved in x g of PEG 400 in 40 mL glass vials. These vials were sealed and subjected to ultrasound irradiation at 50 °C for 20 min to dissolve the polyepoxides. Subsequently, y g of TREN monomers was added to the mixture, and the mixture was ultrasonicated for 10 min at 50 °C. The reaction was then carried out in an oil bath at 110 °C for 3 h without stirring. The reaction of polyamines with polyepoxides in the presence of PEG resulted in the formation of gels with different transparencies, depending on the extent of reaction and phase separation of the porous network during polymer-induced phase separation. After the reaction, the samples were soaked in 200 mL of reverse osmosis-treated (RO) water, then soaked in 200 mL of methanol, and finally soaked in 200 mL of RO water for 24 h at each step. The samples were then freeze-dried for 48 h and labeled as PAEs-X, where X denotes the mole ratio of polyepoxide to polyamine.

### 3.3. Immobilization of Pd Nanoparticles on PAEs

Pd/PAEs-0.94 was synthesized using the conventional impregnation–reduction method. In this process, 100 mg of PAEs-0.94 was dispersed in a 10 mL solution of 1 mg/mL PdCl_2_ in water and stirred for 24 h at room temperature. The resulting solids were washed through centrifugation and then soaked in 20 mL of deionized water for two hours before being centrifuged again. The sample was then dispersed in 10 mL of deionized water and reduced using a freshly prepared 0.01 M NaBH_4_ aqueous solution under vigorous stirring. After 2 h of reduction, the Pd/PAEs-0.94 was obtained by washing the sample three times through centrifugation and then freeze-drying it for 48 h.

### 3.4. Characterization

The Fourier-transform infrared spectrum (FT-IR) of KBr powder-pressed pellets was recorded using a Nicolet iS50 spectrophotometer with a spectral resolution of 4 cm^−1^. Thermal gravimetric analysis was performed using a Mettler TGA/DSC from room temperature to 900 °C at a heating rate of 5 °C min^−1^ under N_2_ gas. The X-ray diffraction (XRD) pattern of the sample was recorded using an X PertPowder. The X-ray tube was operated at 40 kV and 30 mA (Cu Kα radiation with Ni filter, λ = 1.5406 Å). Scans were obtained from 4° to 40° (2θ) at a speed of 1° min^−1^. The elemental analysis (EA) was determined using an Elementar VAROEL III. Inductively coupled plasma (ICP) analysis was performed using a Perkin Elmer Nexion 300. The material stability in common solvents such as dimethyl sulfoxide (DMSO), tetrahydrofuran (THF), and *N*,*N*-dimethylformamide (DMF) was briefly investigated. Approximately 10 mg of PAE samples were added to a vial containing 4 mL of solvent and soaked for 3 days to observe the solubility of the material. The morphologies of PAEs were investigated using scanning electron microscopy (SEM, Zeiss supra55) with an energy-dispersive X-ray spectrometer (EDX). The crystal structure was characterized by transmission electron microscopy (TEM, FEI Tecnai G2 F20) with an operating voltage of 200 kV. Furthermore, N_2_ physisorption measurements (ASAP 2460, Micromeritics, Norcross, GA, USA) were performed on the particles at 77 K to assess their Brunauer–Emmett–Teller (BET) surface areas (SBET) and pore size.

The catalytic performance of Pd/PAEs-0.94 was assessed by means of the reduction of 4-nitrophenol (4-NP) to 4-aminophenol (4-AP) with NaBH_4_, which was monitored with the aid of UV–Vis spectroscopy (Evolution 201, Thermo Fisher Scientific, Waltham, MA, USA). Typically, an excess of 1.5 mL of 14 mmol/L NaBH_4_ was added to 1.5 mL of freshly prepared 0.14 mmol/L 4-NP aqueous solution in a quartz cuvette. Subsequently, 1 mL of 0.4 g/L Pd/PAEs aqueous suspension was introduced into the above mixture at room temperature. The color of the reaction solution underwent a change from light yellow to colorless due to the creation of the reduced 4-AP from 4-NP.

## 4. Conclusions

A novel category of nitrogen-rich porous organic polymers was developed utilizing triamines and triepoxides as raw materials. These synthesized materials were produced through irreversible ring-opening reactions and exhibit both crystalline and amorphous structures, although the degree of crystallinity needs to be increased. The optimal crystalline structure is achieved when the epoxy is equivalent to or in excess of the primary amino group. These porous materials demonstrate exceptional thermal stability and high solvent stability. Moreover, the materials contain a copious amount of secondary amine groups on their skeletal structure and surface, making them suitable for immobilizing guest materials, particularly metal ions. The Pd catalyst was fabricated in situ onto the PAEs support by the NaBH₄ reduction of the Pd precursor, and the size of the nano-Pd was about 6.9 nm. After being impregnated with Pd nanoparticles, these materials exhibited outstanding catalytic performance for the reduction of p-nitrophenol to p-aminophenol. This study presents a new approach for synthesizing porous organic polymers that possess stability, crystallinity, and abundant nitrogen-containing groups, which hold great potential for applications in gas capture, separation, heterogeneous catalysis, and energy storage.

## Figures and Tables

**Table 1:** Demarcating of electron diffraction patterns.

Code	R	d	R^2^/R_i_^2^	N		h	k	l
	/(1/nm)	/nm		Calculated	Rounded			
PAEs-0.94	4.47	0.22	1	1	1	1	0	0
	7.56	0.13	2.87	2.87	3	1	1	0
	8.94	0.11	4.01	4.01	4	2	0	0
	13.42	0.07	9.03	9.03	9	3	0	0
PAEs-1.68	4.44	0.23	1	1	1	1	0	0
	7.27	0.14	2.69	2.69	3	1	1	0
	8.97	0.11	4.09	4.09	4	2	0	0
PAEs-2.06	3.57	0.28	1.08	4.31	4	2	0	0
	4.63	0.22	1.81	7.23	7	2	1	0
	5.29	0.19	2.36	9.45	9	3	0	0
	6.21	0.16	3.26	13.03	13	3	1	0
	7.4	0.14	4.63	18.51	19	3	2	0
	7.87	0.13	5.23	20.91	21	4	1	0
	9.07	0.11	6.95	27.82	28	4	2	0
	10.75	0.09	9.76	39.06	39	5	2	0
PAEs-2.68	2.99	0.33	1	3	3	1	1	0
	3.3	0.3	1.22	3.7	4	2	0	0
	3.34	0.3	1.25	3.7	4	−2	0	0
	4.5	0.22	2.27	6.8	7	−2	−1	0

## Data Availability

Not applicable.
